# Same-session aspiration of unexpected ascites and endoscopic ultrasound-guided gastroenterostomy in gastrojejunal anastomotic stenosis

**DOI:** 10.1055/a-2761-0052

**Published:** 2025-12-19

**Authors:** Alessandro Fugazza, Giacomo Marcozzi, Marta Andreozzi, Gianluca Franchellucci, Matteo Colombo, Marco Spadaccini, Alessandro Repici

**Affiliations:** 1 9268 Division of Gastroenterology and Digestive Endoscopy, IRCCS Humanitas Research Hospital, Rozzano, Milan, Italy; 2 437807 Department of Biomedical Sciences, Humanitas University, Pieve Emanuele, Milan, Italy

Endoscopic ultrasound-guided gastroenterostomy (EUS-GE) is a novel technique for bypassing gastric outlet obstruction through the deployment of a lumen-apposing metal stent (LAMS). Compared with surgical gastrojejunostomy, EUS-GE is associated with fewer adverse events, and compared with enteral stenting, it offers lower recurrence and reintervention rates 1 2 3 . The presence of ascites was traditionally considered a contraindication due to the risk of stent misdeployment 4 . However, recent reports suggest that pre-procedural percutaneous paracentesis can render the procedure technically feasible 5 .

We present the case of a 79-year-old man with pancreatic adenocarcinoma, previously treated with pancreaticoduodenectomy and adjuvant chemotherapy. One year after surgery, he presented with persistent vomiting. Abdominal computed tomography revealed gastrojejunal anastomotic stenosis due to oncologic recurrence, along with a small amount of ascites ( Video 1 ).

EUS-guided gastroenterostomy for malignant gastrojejunal anastomotic stenosis after same-session transgastric aspiration of unexpected ascites to optimize target visualization. Successful LAMS deployment without complications allowed early oral intake. EUS, Endoscopic ultrasound; LAMS, lumen-apposing metal stent.Video 1

EUS-GE was planned but was delayed due to pneumonia. The procedure was then scheduled 10 days later the hospital admission. Intra-procedurally, a large, unexpected rapidly filling perigastric ascites was encountered, impairing adequate visualization of the target jejunal loop. To avoid aborting and rescheduling the procedure which would prolong symptoms, require repeat sedation, and add patient discomfort, we administered prophylactic ceftriaxone and, in the same session, aspirated ~1000 mL of ascitic fluid using a 19-gauge needle via a transgastric approach. This enabled clear visualization of the jejunal loop and safe LAMS deployment, creating gastroenterostomy. The procedure was uneventful, with no immediate or delayed complications. The patient resumed oral intake within few days and was discharged home.

This case highlights the feasibility and safety of same-session EUS-guided ascites aspiration to facilitate EUS-GE in patients with large-volume ascites, potentially avoiding procedural delays, increasing the risk of adverse events and improving patient comfort.

Endoscopy_UCTN_Code_CPL_1AL_2AG

## Notes

Endoscopy E-Videos https://eref.thieme.de/e-videos
*E-Videos* is an open access online section of the journal *Endoscopy* , reporting on interesting cases and new techniques in gastroenterological endoscopy. All papers include a high-quality video and are published with a Creative Commons CC-BY license. Endoscopy E-Videos qualify for HINARI discounts and waivers and eligibility is automatically checked during the submission process. We grant 100% waivers to articles whose corresponding authors are based in Group A countries and 50% waivers to those who are based in Group B countries as classified by Research4Life (see: https://www.research4life.org/access/eligibility/ ). This section has its own submission website at https://mc.manuscriptcentral.com/e-videos .
